# A Novel Electro-Thermal Laminated Ceramic with Carbon-Based Layer

**DOI:** 10.3390/ma10060641

**Published:** 2017-06-12

**Authors:** Yi Ji, Bin Huang, Pinggen Rao

**Affiliations:** 1 School of Materials Science and Engineering, South China University of Technology, Guangzhou 510640, China; 18771021089@163.com; 2 Guangdong Industry Technical College, Guangzhou 510300, China; Binoo@126.com

**Keywords:** laminated ceramics, carbon-based layer, electro-thermal property, adhesive strength, heat supply

## Abstract

A novel electro-thermal laminated ceramic composed of ceramic tile, carbon-based layer, dielectric layer, and foaming ceramic layer was designed and prepared by tape casting. The surface temperature achieved at an applied voltage of 10 V by the laminated ceramics was 40.3 °C when the thickness of carbon-based suspension was 1.0 mm and the adhesive strength between ceramic tile and carbon-based layer was 1.02 ± 0.06 MPa. In addition, the thermal aging results at 100 °C up to 192 h confirmed the high thermal stability and reliability of the electro-thermal laminated ceramics. The development of this laminated ceramic with excellent electro-thermal properties and safety provides a new individual heating device which is highly expected to be widely applied in the field of indoor heat supply.

## 1. Introduction

Conductive composites have been a new functional material since 1950 due to their outstanding conductivity, stability, and heat resistance [1]. Carbon-based composites have additional advantages of high thermal efficiency, low cost, and light weight [2,3]. These combined characteristics are essential prerequisites for their application of indoor heating devices and electromagnetic/radio frequency interference (EMI/RFI) shielding [4,5,6]. In the field of indoor heating, about 2% heat sources come from individual heating facilities [7]. For example, the eco-friendly electro-thermal films based on conductive composite have been gradually applied to indoor heat supply in cold areas. However, the air-gap between the floor substrate and electro-thermal films causes thermal dissipation. The installation of electro-thermal films is also a complicated procedure.

Many studies show the growing interest towards hybrid fillers for conductive composites [8,9]. The effects of types, morphologies, and particle sizes of fillers on the electrical and thermal performances of conductive composites have been addressed by many researchers [10,11]. Since the observation of carbon nanotubes [12] and graphene [13], an explosion of interest has been focused on the electrical, thermal, and mechanical properties of the conductive nanocomposites consisting of the two novel carbon materials [14,15,16,17]. However, no experimental work has been reported on the application of conductive composites in the field of indoor heating devices. In order to solve the air-gap problem and develop a new individual heating device with high reliability and electro-thermal property at low voltages, preparing a laminated ceramic with an electro-thermal layer is a promising approach due to its safety, high thermal efficiency, aesthetics, and integrality.

In this paper, the novel electro-thermal laminated ceramics composed of ceramic tile, carbon-based layer, dielectric layer, and foaming ceramic layer were designed and prepared. The effects of applied voltage and carbon-based suspension thickness on the surface temperature of the laminated ceramics were studied. The interfacial adhesion between ceramic tile and carbon-based layer was then characterized. In addition, a comparative study of adhesive strength and electrical resistivity during thermal aging at 100 °C was conducted to analyze the thermal stability of the laminated ceramics.

## 2. Materials and Methods

Carbon-based layers were the composites of conductive fillers and water-based acrylic resin matrix (WA-2007A, Jelee, Zhuhai, China). Graphite (99.8%, purity), carbon black, and nickel (99.9%, purity) with a mass ratio of 45.5 wt %; 43.5 wt %; 11.0 wt % were used as conductive fillers. Firstly, 55 wt % conductive fillers and 45 wt % resin solution were mixed by ball milling (QM-3SP2, Nanjing University Instrument Factory, Nanjing, China) at 400 rpm/min for 1 h. Secondly, the carbon-based layers (20.5 cm length × 2.0 cm width) were prepared by spreading the carbon-based suspensions on the ceramic tiles (5.0 mm thickness) by tape casting with the automatic film applicator (BEVS1811, BEVS, Guangzhou, China) at a rate of 20 mm/s and then dried at room temperature. The thickness of the suspension applied on the ceramic tile was adjusted by changing the blade gap using micrometric screws. Thirdly, the conductive silver paste used as electrode material was spread on the two ends of the carbon-based layer and then the conductive copper tapes were pasted on to be used as wires. Subsequently, the silicone sealant was coated on the dry carbon-based layer as a dielectric layer, on which the foaming ceramics (3.0 cm thickness) with a thermal conductivity of 0.15 W/(m·K) were bonded as thermal-insulated material.

The morphology of the laminated ceramics was observed by scanning electron microscopy (SEM, EVO18, Zeiss, Jena, Germany). The DC power supply (MPS-6003S, Matrix, Shenzhen, China) was used to apply voltage on samples. The surface temperature of the laminated ceramics was tested by digital thermometer (TM-902C, BRD, Shanghai, China). According to the ISO 4624:2002 standard, the adhesive strength between the ceramic tile and the carbon-based layer can be determined. One cylindrical metal mold was glued on the surface of the carbon-based layer using epoxy glue. After curing, the adhesive strength was measured using a tensile equipment (AT-A, DeFelsko, New York, NY, USA). The electrical resistivity of the carbon-based layer was characterized by a four probe instrument (ST2263, Jingge, Suzhou, China).

## 3. Results and Discussion

**Figure 1:**
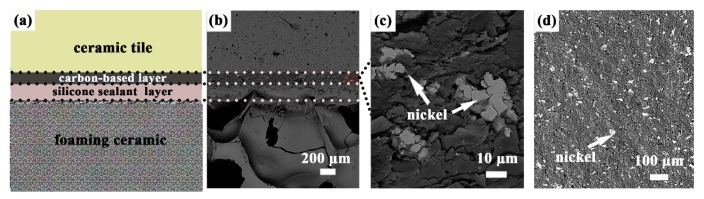
(**a**) Structure illustration of the laminated ceramics; (**b**) cross-section backscattering electron image of the laminated ceramics; (**c**) cross-section backscattering electron image of the carbon-based layer; (**d**) surface backscattering electron image of the carbon-based layer.

Figure 1a shows that the laminated ceramics are composed of ceramic tile, carbon-based layer, silicone sealant layer, and foaming ceramic layer successively. The cross-section backscattering electron image in Figure 1b displays the dense interface without air-gaps between ceramic tile and carbon-based layer. Cross-section backscattering electron image and surface backscattering electron image of the carbon-based layer are shown in Figure 1c,d, respectively. Obviously, the nickel particles are homogenously dispersed in the carbon-based layer and play an important role in bridging the neighboring graphite particles to form more conductive pathways and then improve the conductivity of carbon-based layer further. Therefore, the electro-thermal properties of the laminated ceramics should be attributed to the three-dimensional conductive network constructed by the conductive fillers in the carbon-based layer.

**Figure 2:**
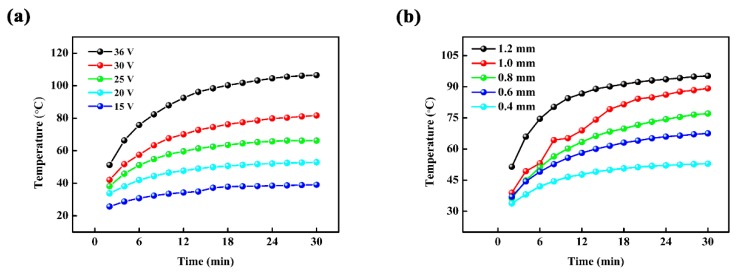
The effects of (**a**) applied voltage and (**b**) carbon-based suspension thickness on the surface temperature of the laminated ceramics.

Figure 2a represents the effect of applied voltage on the surface temperature of the laminated ceramics with carbon-based suspension thickness of 0.4 mm. It can be seen that the surface temperature increases from 39.1 °C to 106.5 °C with applied voltage from 15 V to 36 V for 30 min. The required temperature of indoor heat supply (around 40–50 °C) has been obtained for the laminated ceramics at 20 V already. It is also found that temperature distribution on the surface of the laminated ceramics is quite uniform. This may be attributed to the well-distributed conductive fillers in the carbon-based layer and the consistent thickness of carbon-based layer controlled by tape casting. In addition, the influence of the carbon-based suspension thickness on the surface temperature of the laminated ceramics at 20 V is shown in Figure 2b. The surface temperature of the laminated ceramics increases with the thickness of carbon-based suspension and reaches up to 95.4 °C when the thickness is 1.2 mm. It can be explained by the equation

$$R=\frac{\rho \times L}{\sigma \times d}$$

where *R* is the electrical resistance, *ρ* is the volume resistivity, *L* is the length, *σ* is the thickness, and *d* is the width, respectively. The electrical resistance of carbon-based layer reduces with the increase of thickness and then the temperature increases with the decrease of electrical resistance at a constant applied voltage according to the Joule’s law.

**Figure 3:**
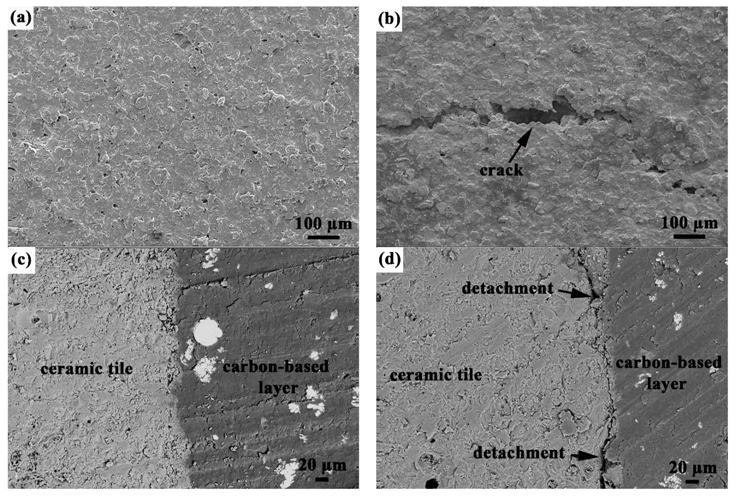
Surface SEM images of the carbon-based layer with suspension thickness of (**a**) 1.0 mm and (**b**) 1.2 mm. Interface backscattering electron images between ceramic tile and carbon-based layer with suspension thickness of (**c**) 1.0 mm and (**d**) 1.2 mm.

Figure 3a,b show the SEM images comparing the surface morphology of the carbon-based layer with suspension thickness of 1.0 mm and 1.2 mm. Compared with the unbroken surface of the 1.0 mm sample shown in Figure 3a, some cracks can be clearly observed on the 1.2 mm sample as indicated by the arrow in Figure 3b. Besides, Figure 3c is a typical backscattering electron image showing the good interfacial adhesion between ceramic tile and carbon-based layer with suspension thickness of 1.0 mm. However, in the case of the 1.2 mm sample, partial detachment from the ceramic tile is detected as indicated by the arrows in Figure 3d. Correspondingly, the adhesive strength of the carbon-based layer with suspension thickness of 1.0 mm and 1.2 mm is 1.02 ± 0.06 MPa and 0.95 ± 0.09 MPa, respectively. This may be attributed to the accumulated internal residual stress as the layer is thickened, which causes defects on the surface and a reduction in the adhesive strength [18].

**Figure 4:**
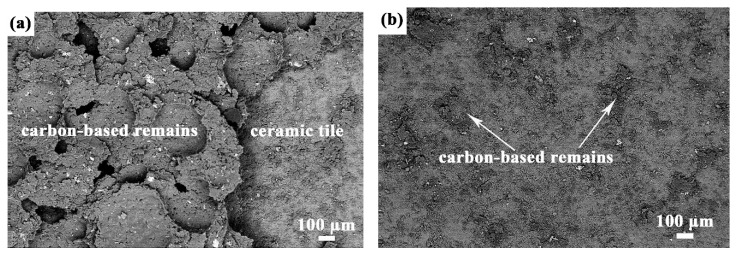

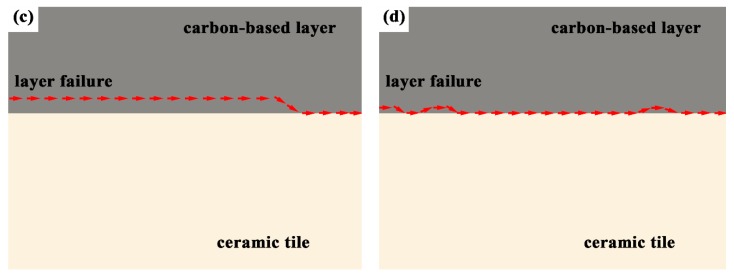
Backscattering electron images of the fracture surface of the samples with carbon-based suspension thickness of (**a**) 1.0 mm and (**b**) 1.2 mm after tensile adhesion strength test. Schematic diagrams of the failure mode of the (**c**) 1.0 mm samples and (**d**) 1.2 mm samples.

Figure 4a,b show the morphology of the ceramic tiles after the carbon-based layers with suspension thickness of 1.0 mm and 1.2 mm are pulled off, respectively. Obviously, a large amount of the carbon-based remains can be observed on the ceramic tile in Figure 4a. It is indicated that the cohesive failure effectively contributes to the fracture, as shown in Figure 4c. As comparison, from backscattering electron image of the 1.2 mm sample in Figure 4b, the fracture surface implies that the layer failure mainly occurs between carbon-based layer and ceramic tile. Although adhesive failure dominates the fracture, some cohesive features evidenced by the small amount of carbon-based remains on the ceramic tile can be observed, as indicated by the arrows in Figure 4b. Similarly, the failure mode of the 1.2 mm samples is shown in Figure 4d.

**Figure 5:**
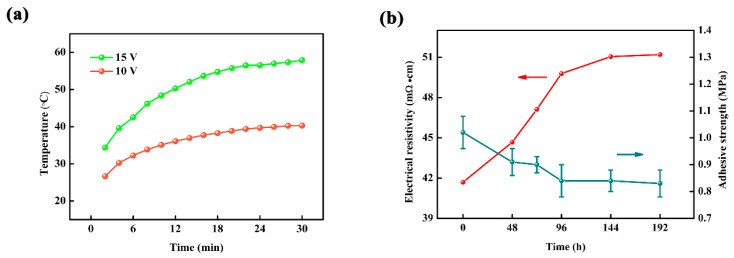
(**a**) The effect of applied voltage on the surface temperature of the laminated ceramics with carbon-based suspension thickness of 1.0 mm; (**b**) adhesive strength and electrical resistivity of the carbon-based layer with suspension thickness of 1.0 mm after aging at 100 °C up to 192 h.

Figure 5a depicts the surface temperature of the laminated ceramics with carbon-based suspension thickness of 1.0 mm at the applied voltage of 10 V and 15 V. It can be seen that the surface temperature of the laminated ceramics at 10 V for 30 min is 40.3 °C, which means much more energy-saving available for indoor heat supply. The thermal stability analysis results after aging at 100 °C up to 192 h are given in Figure 5b. We can see that the adhesion strength decreases from 1.02 ± 0.06 MPa to 0.83 ± 0.05 MPa, which is caused by the degeneration of the mechanical bond between ceramic tile and carbon-based layer. Meanwhile, the electrical resistivity increases from 41.70 mΩ·cm to 51.20 mΩ·cm. The possible reason for this is that the thermal expansion coefficient of resin matrix is higher than that of metal and carbonic fillers. Therefore, the expansion of resin matrix increases the distance between conductive fillers and destroys the original conductive network during aging, resulting in the reduction of conductivity [19]. However, the adhesive strength and electrical resistivity tend to be stable after aging for 96 h, suggesting that the laminated ceramics possess good thermal stability and reliability, which are crucial for them to be applied to indoor heat supply.

## 4. Conclusions

In summary, the novel electro-thermal laminated ceramics composed of ceramic tile, carbon-based layer, silicone sealant layer, and foaming ceramic layer were prepared successfully. The electro-thermal properties of the laminated ceramics are attributed to the good conductivity of the homogeneously dispersed conductive fillers in the carbon-based layer and the excellent thermal insulation effect of the foaming ceramics. Moreover, the laminated ceramics exhibit safety, thermal stability, and reliability as well as good electro-thermal properties at low voltages. As an ideal individual heating device, the electro-thermal laminated ceramics are highly expected to be widely applied in the field of indoor heat supply.
